# Implementation of Telepharmacy to Provide Medication Consultation Service for Patients: A Case Study From Thu Duc City Hospital, Ho Chi Minh City

**DOI:** 10.7759/cureus.60746

**Published:** 2024-05-21

**Authors:** Truong Van Dat, Nguyen Hong Minh, Thai Minh Hoang, Tran Thu Hien, Phan Nguyen Hoai Bao, Vo Linh Tu, Nguyen Vu Hung, Nguyen Huu Lac Thuy, Dang Thi Kieu Nga

**Affiliations:** 1 Saigon Pharmaceutical Science and Technology Center, University of Medicine and Pharmacy at Ho Chi Minh City, Ho Chi Minh, VNM; 2 Ministry Office, Ministry of Health, Vietnamese, Ha Noi, VNM; 3 Faculty of Pharmacy, University of Medicine and Pharmacy at Ho Chi Minh City, Ho Chi Minh, VNM; 4 Faculty of Pharmacy, Thu Duc City Hospital, Ho Chi Minh, VNM; 5 Faculty of Traditional Medicine, University of Medicine and Pharmacy at Ho Chi Minh City, Ho Chi Minh, VNM

**Keywords:** survey, hospital, thu duc city, medication consultation service, telepharmacy

## Abstract

Telepharmacy is receiving significant attention as an innovative approach. The objective of this study is to assess the needs and evaluate the impact of telepharmacy applications in drug consultations at Thu Duc City Hospital. We used a cross-sectional research design and conducted a survey with the participation of leaders of the Faculty of Pharmacy, clinical pharmacists, dispensing pharmacists, and patients or their caregivers who receive medication at the Pharmacy of Thu Duc Hospital. We deployed a telepharmacy application for consulting on drug use and surveyed the satisfaction of patients/family members with the telepharmacy model. 60.3% of survey subjects expressed a desire to receive drug use consultations through telepharmacy if the hospital were to offer this service. One hundred percent of the pharmacists at the pharmacy and the hospital's leadership believe that telepharmacy can address patient consultation needs and improve the current physical facilities in the dispensing area. Over 90% of telepharmacy users reported being satisfied or very satisfied with the service. Telepharmacy has garnered the attention of patients, their caregivers, and the medical staff at the Outpatient Pharmacy of Thu Duc Hospital. The majority of users are satisfied with the drug use consultation service provided by telepharmacy. By expanding the good results of Thu Duc Hospital to other hospitals, more patients across Vietnam can benefit from this innovative approach.

## Introduction

Amidst the Fourth Industrial Revolution, telepharmacy (remote pharmaceutical care) has gained considerable attention [1,2]. This attention has been further accentuated by the challenges posed during the COVID-19 pandemic, which mandated movement restrictions and stringent physical distancing measures, leading to a surge in healthcare demand. Telemedicine, regarded as a paramount method of care support, facilitates electronic consultations while reducing the risk of disease transmission by minimizing direct contact [3]. Many healthcare agencies and policymakers have rallied behind the adoption of telemedicine as a means to confront the challenges facing the healthcare system during the COVID-19 crisis. Telepharmacy, much like its telemedicine counterparts, has garnered increased attention in the wake of the COVID-19 pandemic [4]. Telepharmacy stands as a practical component of telemedicine, involving the provision of pharmaceutical services under the guidance of pharmacists while maintaining spatial and temporal distance. This practice embraces physical distancing between patients, health service users, and healthcare professionals [5]. For the first time in the world, regulations related to the use of telepharmacy were put into law in the state of North Dakota, USA, in 2001. Today, telepharmacy activities are being widely applied globally [6-8]. Many countries have even included this activity in their health policy, such as the United States, China, Australia, Spain, Denmark, etc., to increase access to care services in special geographical areas. In addition, it can also reach some patients or facilities that do not have enough professional resources on-site [3,9-13].

Thu Duc City Hospital was recognized as a first-class district hospital in Vietnam. At present, the hospital has fully deployed high-tech specialties by faculties. Furthermore, the hospital receives and performs an average of 5,000-6,000 outpatients and more than 200 emergency cases every day. As the number of patients accessing the hospital each day is increasing, the hospital has pioneered the implementation of remote medicine consultation services. Although our previous survey conducted from February to July, 2021, showed that in Ho Chi Minh City, 87.2% of participants were willing to apply telepharmacy in pharmacy practice, that means 361 out of 414 pharmacists were ready to use this service [14]. This activity is still very new and has not been widely deployed; besides, there are also no related regulations and guidelines. The dispensing counter at Thu Duc City Hospital receives 600-800 visitors per day, mainly focusing on supply and dispensing activities. Although providing drug information to patients is one of the pharmacist's tasks, consulting on drug use at the counter is actually only done through the "hotline" phone number of the Clinical Pharmacy team. The purpose is for the patient or family to contact whenever needed. While phone consultations offer convenience, they lack the visual and dynamic elements of in-person interactions. They are primarily suited for addressing simple inquiries from patients or their families regarding the dosage or usage of specific medications. The objective of this study is to survey the current situation and need for consultation on drug use at the Pharmacy at Thu Duc City Hospital. It further seeks to develop the implementation process, deploy the initiative, and evaluate the initial results of applying information technology in consulting on drug use at the Pharmacy of Thu Duc City Hospital.

## Materials and methods

Survey method

A case study was conducted at Thu Duc City Hospital Pharmacy, from March 2022 to September 2022. The Medical Ethics Council at Thu Duc City Hospital issued approval 26/BV-HDDD. The hospital established the necessary technological infrastructure, including secure communication channels and software platforms compliant with patient data protection regulations. Pharmacists received specialized training on conducting remote consultations effectively, ensuring they were proficient in using the telecommunication tools and adhering to ethical guidelines. Information about the availability and benefits of telepharmacy services was disseminated through various channels, such as social media platforms and brochures.

The subjects included the Board of Directors of the pharmacy department, clinical pharmacists, dispensing staff at the pharmacy, as well as patients or family members receiving medicine at the Pharmacy. The sampling criteria were as follows: All patients or family members who received medicine at the Pharmacy after an outpatient examination, agreed to participate in the survey, and were 18 years old or older. The exclusion criteria were emergency patients, patients given Special Control drugs, patients or family members who did not agree to participate in the survey, and those who did not complete the survey.

The survey form included: (1) A consent form to participate in the study; (2) A patient/family survey form on the willingness to use telepharmacy services; (3) A survey form for the Board of Directors, Clinical Pharmacists, and Pharmacy staff; (4) A patient/family experience survey form after receiving consultation on medication use through telepharmacy. We conducted a survey of patients/patient caregivers at Thu Duc City Hospital Pharmacy to find out their interest in telepharmacy services.

The content covered the willingness to use remote medication counseling services and the experience of telepharmacy services. Survey forms were also distributed to the Board of Directors, Head of Pharmacy Department, Clinical Pharmacists, and Pharmacy staff. During the survey on consultation needs and responses related to consultation via telepharmacy, the researcher asked additional questions about the patient's disease or medication use. Simultaneously, patients/caregivers were instructed on how to install the Telme application. A schedule was set to receive contact from the patient. The consultation process was designed based on general instructions on implementation steps, and consultation content provided by the American Pharmacists Association in 2019 [15,16] as well as the Patient Counseling chapter from the Clinical Pharmacy Practice Book [17]. Additionally, further drug information requirements were specified in Pharmacy Law 105/2016.

The study evaluated the initial results of applying information technology to medication consultation at the Pharmacy of Thu Duc City Hospital by surveying the experiences of patients/family members who received medication consultation via Telepharmacy.

Data processing

Data were entered using Microsoft Excel and analyzed with Statistical Package for Social Sciences (SPSS) version 25 statistical software (IBM Corp., Armonk, NY, USA). Continuous variables with normal distribution and homoscedasticity were presented as mean ± standard deviation. Continuous variables with non-normal distribution and/or heterogeneous variance were presented using median and interquartile range. Categorical variables are described by number and percentage. The Chi-square test was used to compare proportions between groups with 95% confidence (α = 0.05).

## Results

Results of the willingness to use telepharmacy services

During the research period from March 2022 to August 2022, we collected 697 subjects who met the sampling criteria. Of these, 33 samples were eliminated due to not completing the survey or being under 18 years old without a family member present. Among the subjects meeting the criteria, 496 (71.2%) wanted advice on medication use, and 201 (28.8%) refused further advice. When asked about their willingness to use telepharmacy services, 420 (60.3%) agreed, and 277 (39.7%) refused (Table 1).

**Table 1:** General characteristics of the population and interest in telepharmacy services (n = 697) *p<0.05 is considered significant

Characteristics	Interest in telepharmacy services				Total		P-value*
	Yes		No				
	N	%	N	%	N	%	
Survey subjects							
Patient	286	56.5	220	43.5	506	72.9	p = 0.001
Patients' relatives	134	70.2	57	29.8	191	27.1	
Patient age group							
Under age 6	69	69.7	30	30.3	99	14.2	p = 0.058
From 6 to 18	31	68.9	14	31.1	45	6.5	
From 18 to 30	77	65.8	40	34.2	107	16.8	
From 30 to 44	86	56.6	66	43.4	152	21.8	
From 44 to 60	84	56.8	64	43.2	148	21.2	
Over age 60	73	53.7	63	46.3	136	19.5	
Initial examination/re-examination							
Initial examination	199	62.0	122	38.0	321	46.1	p = 0.387
Re-examination	221	58.8	155	41.2	376	53.9	
Level of support during examination							
Good	290	57.5	214	42.5	504	72.3	p = 0.006
Medium	18	51.4	17	48.6	35	5.0	
Least	112	70.9	46	29.1	158	22.7	
Ability to self-administer medication							
Good	288	57.8	210	42.2	498	71.4	p = 0.038
Least	132	66.3	67	33.7	199	28.6	
Forgetting to take medicine on time							
Yes	199	61.6	124	39.4	323	46.3	p = 0.498
No	221	59.1	153	40.9	374	53.7	

In the research sample, the direct subjects interviewed for the survey were patients or accompanying family members. Patient ages ranged from two months to 88 years, with a median age of 38 years. The rate of patients having re-examination (including regular re-examinations for chronic diseases and re-examinations by appointment) is higher than that of subjects going for examination for the first time. However, the difference in demand for telepharmacy consultation is not statistically significant. Factors such as survey subjects, the level of need for assistance during examination, and the ability to self-administer medication showed statistically significant differences (p = 0.001; 0.006; 0.038).

**Table 2:** Reasons for not being willing to use telepharmacy services of 277 participants

Reasons for not being willing to use telepharmacy services	N (%)
It feels complicated and the form is new so I don't want to try it	52 (18.8%)
Busy and don't have time	47 (17.0%)
It seems unnecessary or lacking in need	42 (15.2%)
The doctor has given advice, if necessary, I will ask the doctor	32 (11.6%)
Do not use smart devices	14 (4.9%)
Other reasons	28 (10.3%)
Refuse to answer	77 (27.7%)

Among the answers, 52 participants assumed that the most common reasons for refusing to participate in medication consultation via telepharmacy are feeling that the process is complicated and the format is new (18.8%), and 47 of them said they did not have time (17%).

Results of an actual survey of consulting on medication consultation at the Outpatient Pharmacy of Thu Duc Hospital

We surveyed one doctor from the Hospital Board of Directors responsible for operational management, one pharmacist in charge of the professional team at the Pharmacy Department, one pharmacist in charge of the Dispensing Counter, one clinical pharmacist, and 22 pharmacists dispensing at the counter. The survey results are presented in Table 3.

**Table 3:** Survey on the need for consultation on medicine use from the perspective of the dispensing counter staff

Questions	Answers	N (%)
Consulting on medication use is necessary for patients?	Yes	26 (100.0%)
	No	0 (0.0%)
Who specifically receives benefits from consulting?	Elderly people (>= 60 years old)	14 (53.8%)
	Patients with liver/kidney failure	5 (19.2%)
	Pregnancy/ Breastfeeding women	2 (7.7%)
	All special subjects	5 (19.2%)
Which is the current status of the need for consultation on medicine use at the Dispensing Counter? (answered by clinical pharmacist, pharmacist at the Counter)	Less than 10% of patients/family members need advice on medication use	6 (25%)
	More than 50% of patients/family members need consultation on medication use	5 (20.8%)
	About 10 - 50% of patients/family members need consultation on medication use	13 (54.2%)
Medication consultation should be done by which medical staff?	Pharmacist of Pharmacy Department	21 (80.8%)
	Doctor: person who directly examines patients	4 (15.4%)
	Nursing in a specific department	1 (3.8%)
	Any medical staff, appropriately arranged according to human resource conditions	4 (15.4%)

There were 26 (100%) survey subjects who stated that counseling on medication use is necessary for patients. When asked about which specific patients benefit most from medication consultation, more than 80% indicated the elderly (≥ 60 years old). Based on practical experience dispensing at the counter, the majority of pharmacists (78%) believed that less than 50% of patients require advice on drug use. Additionally, 100% of survey subjects affirmed that telepharmacy can be a solution to the need for consultation on medicine use at dispensing counters. The recorded reasons include: helping patients receive more professional advice; minimizing direct contact with patients with infectious diseases; pharmacists participating throughout the drug use process, thereby reducing related costs; solving the shortage of pharmacists; caring for remote areas; and addressing the lack of space and time for on-site consulting.

Evaluating the impact of telepharmacy application in medication consultation

We provided medication consultation via telepharmacy for 53 cases. Since this is the first study in Vietnam to apply telepharmacy to remotely counsel on medicine use for patients, the intervention aims to supplement counseling and improve knowledge and compliance with the medication of patients. However, due to technological barriers, smartphone usage habits, and patient age, the rate of patients participating in consultation is not high compared to the rate of consent. In the study sample, the median age was 32 (± 8.4) years, with the lowest being 18 and the highest being 44. We conducted a survey on the satisfaction level of patients/family members regarding the overall consultation process and the answers to their questions, using a 5-point scale: 1 point - very dissatisfied or very poor, 2 points - dissatisfied or poor, 3 points - normal or average, 4 points - satisfied or good, 5 points - very satisfied or very good. The results are presented in Table 4.

**Table 4:** Survey of experiences after telepharmacy on medication consultation

Questions	Answer	N (%)
Were your initial questions answered?	Yes	53 (100.0%)
	No	0 (0.0%)
Are you satisfied with the answers or professional advice provided by the Pharmacist?	3 - Normal	3 (5.7%)
	4 - Satisfied	5 (9.4%)
	5 - Very satisfied	45 (84.9%)
Are you satisfied with the pharmacist's attitude?	3 - Normal	3 (5.7%)
	4 - Satisfied	5 (9.4%)
	5 - Very satisfied	45 (84.9%)
Do you believe and follow what you are advised?	Yes	53 (100.0%)
	No	0 (0.0%)
Do you wish to continue consulting on medication use by telepharmacy at your next visit?	Would like to continue the service	39 (73.6%)
	Consider using the service if needed, depending on the next visit	12 (22.6%)
	Prefer direct consultation	2 (3.8%)
For you, what are the advantages of telepharmacy?	Convenient, feasible to schedule a consultation and arrange time	36 (67.9%)
	Limit direct communication to avoid the risk of spreading the disease	16 (30.2%)
	Enthusiastic pharmacist	33 (62.3%)
	Useful consultation content, answers to questions about diseases or medications	40 (75.5%)
	Increase the trust and peace of mind when using medicine	9 (17.0%)
Do you have any suggestions to improve Telepharmacy services?	Call volume is low, needs improvement	1 (1.9%)
	Transmission quality affects service quality	1 (1.9%)

Patients/family members who were surveyed after counseling on medication use all gave positive feedback about their service experience. They believe that all questions related to disease/medication use meet the wishes of the person being consulted and are completely confident in following the advice. Overall satisfaction score of the answers or professional advice provided by the pharmacist and the overall score stands for satisfaction with the pharmacist's attitude were 4.79. As can be seen from the results, over 90% of the study subjects were satisfied with the professional consulting content and attitude of the consulting pharmacist and considered continuing to use telepharmacy consulting services. When they were asked more about the reason for wanting to continue using the service, more than 60% of users found this service convenient for scheduling their time, noting the enthusiasm of the consulting pharmacist. As a result, they evaluated the consulting content favorably.

## Discussion

In the study population, 496 participants (71.2%) needed advice on drug use, while the remaining 201 (28.8%) did not need advice. When asked about their willingness to use telepharmacy for medication consultation, only 420 (60.3%) from the survey sample wanted to participate, a decrease of about 10% compared to the need for medication consultation. Some limitations of the new form of implementation, such as equipment and transmission requirements, may hinder patient/caregiver acceptance of participation. In the group that refused to participate in medication consultation by telepharmacy, the common reasons for refusal were feeling uncomfortable with the new form and not wanting to use it, according to 52 participants (18.8%); and not having enough time, according to 47 participants (17.0%). The reason "having been fully consulted by a doctor" was mentioned by only 32 (11.6%). Patients are unfamiliar with or uncomfortable using technology and are concerned about the security and privacy of online interactions. These drawbacks have been previously pointed out by some studies [18-21]. This may explain why some people may not be aware of telepharmacy services or their benefits. Therefore, publicizing or promoting telemedicine services is necessary.

The positive point in the initial steps of implementing telepharmacy is that over 90% of users felt satisfied or very satisfied with the service; everyone completely followed the advice on medication consultation. This indicates that the initial implementation was well-received by the users. Complete adherence to medication consultation advice by all users suggests that the telepharmacy service effectively conveyed information and instructions related to medication usage. Telepharmacy provides convenience by enabling users to receive pharmacy services from the comfort of their homes or preferred locations [18]. This can save time and reduce the hassle associated with traditional pharmacy visits. Thirty-nine (73.6%) users wanted to continue receiving telepharmacy for their next medication pick-up. There were only two users (3.8%) who, after using the service, wanted direct consultation for the next time they took medicine. This rate is quite low compared to Margusino-Framiñán et al.'s study in Spain in 2021, where 55.9% of users wanted to receive medicine and consultation at the hospital if it was convenient to go to the hospital to see a doctor [18]. The positive feedback indicates a high level of satisfaction and trust in the telepharmacy experience. Users likely appreciated the convenience, accessibility, and personalized care offered through telepharmacy consultations. This suggests that the vast majority found telepharmacy to be an effective and satisfactory alternative to in-person consultations. However, the preferences of these users should still be accommodated to ensure they receive the care they need. It's essential to address the preferences of the small minority who prefer direct consultation. Understanding their reasons for dissatisfaction can help improve the telepharmacy service and ensure that all users have a positive experience.

This difference may be due to differences in sample size, age, and occupational status of the two studies. Among 53 answers, regarding the benefits of telepharmacy, the most mentioned were convenience and the ability to arrange their time, declared by 36 (67.9%) and useful consulting content, helping users answer questions about diseases or medications by 40 (75.5%). Telepharmacy can help people manage their medications more effectively [4], leading to better health outcomes [6]. Telepharmacy patients often report higher levels of satisfaction with their care than patients who receive traditional in-person care [18]. Overall, telepharmacy offers a number of benefits that can make it a valuable option for people who need healthcare services. Thus, the new form of consultation with both pharmacists and patients/caregivers, and the positive reception of users, suggests wider implementation of this form of implementation. Additionally, patients value the opportunity to discuss their medication concerns with knowledgeable pharmacists, who provide personalized guidance and recommendations.

Our research shows that Thu Duc Hospital in Vietnam has successfully implemented telepharmacy. By expanding the good results of Thu Duc Hospital to other hospitals, more patients across Vietnam can benefit from this innovative approach. It would be beneficial for healthcare providers and policymakers to collaborate and share best practices in implementing telepharmacy, ensuring its successful adoption and widespread use throughout the country.

There are some limitations to this research including the lack of screening those in need and increase in the level of access to telepharmacy for these subjects. Besides, the target sample size is a bit small (only 53 samples), so it is not possible to completely evaluate the patients’ experience with the software or compare the effectiveness with the traditional form (in physical). This is a pioneering study, so there are no comparative models to accurately evaluate the effectiveness. The initial implementation at a level 1 hospital may not be representative of the effectiveness of nationwide application. Therefore, it is necessary to continue researching and implementing telepharmacy in many different hospitals and areas with a bigger sample size in order to obtain a more objective and comprehensive assessment of the effectiveness of this model.

## Conclusions

Telepharmacy receives the attention of patients/patients' families and medical staff at Thu Duc Hospital. Most people who experience consultation on medication consultation by telepharmacy feel satisfied with the service. As telepharmacy continues to evolve, it is likely to play an increasingly important role in transforming healthcare services and improving patient outcomes.
